# Reducing the receipt of contraindicated medications in patients with Parkinson disease

**DOI:** 10.3389/fnagi.2023.1271072

**Published:** 2023-10-12

**Authors:** Caroline Goldin, Stefan Sillau, Elisa Worledge, Jarrett Bremmer, Robbie Cummins, Kathryn Tremolet de Villers, Michelle E. Fullard

**Affiliations:** ^1^Department of Neurology, Ochsner Medical Center, New Orleans, LA, United States; ^2^Department of Neurology, University of Colorado, Aurora, CO, United States; ^3^University of Colorado Skaggs School of Pharmacy and Pharmaceutical Sciences, Denver Health, Denver, CO, United States; ^4^Baptist Health, Jacksonville, FL, United States; ^5^Department of Pharmacy, University of Colorado Hospital, Aurora, CO, United States

**Keywords:** Parkinson ‘s disease, best practice alert, quality improvement, electronic medical record, contraindicated medications

## Abstract

**Background:**

The administration of antidopaminergic medications to patients with Parkinson’s disease (PD) can exacerbate symptoms, and in the hospital setting, can lead to complications and increased length of stay. Despite efforts to improve medication administration through provider education and patient-centered interventions, the problem persists, with an estimated 21–43% of hospitalized PD patients receiving dopamine blocking medications.

**Methods:**

In this study, a best practice alert (BPA) was developed that was triggered when an antidopaminergic medication was ordered in the Emergency Department or hospital for a patient with a diagnosis of PD in the EMR. The primary outcomes were receipt of a contraindicated medication, length of stay (LOS) and readmission within 30 days. These outcomes were compared between the 12 months prior to the intervention and the 12 months post intervention. Data were also collected on admitting diagnosis, admitting service, neurology involvement and patient demographics.

**Results:**

For pre-intervention inpatient encounters, 18.3% involved the use of a contraindicated medication. This was reduced to 9.4% of all inpatient encounters for PD patients in the first 3 months post-intervention and remained lower at 13.3% for the full 12 months post-intervention. The overall rate of contraindicated medication use was low for ED visits at 4.7% pre-intervention and 5.7% post-intervention. Receipt of a contraindicated medication increased the risk of a longer length of stay, both before and after the intervention, but did not significantly affect 30-day readmission rate.

**Conclusion:**

An EMR BPA decreased the use of contraindicated medications for PD patients in the hospital setting, especially in the first 3 months. Strategies are still needed to reduce alert fatigue in order to maintain initial improvements.

## Introduction

Parkinson Disease (PD) is a neurodegenerative disorder caused by loss of dopamine-producing neurons in the substantia nigra. Further depletion of dopamine with dopamine antagonists, such as antipsychotics and antiemetics, can lead to worsening PD symptoms, cognitive changes, falls, and infections (Chou et al., 2011). In hospitalized patients, receipt of contraindicated medications has been shown to increase length of hospital stay (Chou et al., 2011). In addition, PD patients who are hospitalized are more susceptible to hallucinations, mental status changes, and nausea, symptoms that are typically treated with anti-dopaminergic medications. These patients often have complicated medication regimens, and inpatient staff may be unfamiliar with the management of this largely outpatient-treated disease. In one study, 70% of inpatient staff were unaware of which medications to avoid in patients with PD (Chou et al., 2011). Due to these challenges, contraindicated medications are often inadvertently prescribed to patients with PD who are admitted to the hospital. Studies in hospitals throughout the US and beyond demonstrate that 21–43% of hospitalized PD patients received dopamine-blocking medications, which was associated with complications and longer hospital stays (Derry et al., 2010; Oguh, 2012).

Several measures could be taken in attempts to improve medication administration to PD patients. Patient and provider education on contraindicated medications in PD is important. While providers may immediately better understand the risks associated with administering certain medications to PD patients, the disconnect between a didactic session and hands-on patient care that may occur months after the session can hamper retaining of the information. The Parkinson’s Foundation put out the “Aware in Care” kit, which provides information for patients to hand out to staff members during a hospitalization. This includes the importance of medication timing and a list of contraindicated medications and encourages the patient or care partner to be an advocate. While these kits are certainly helpful, without other interventions, they put the responsibility on the patients and their families to keep this information with them and to recognize when a medication should not be given; efficacy depends on the involvement of the patient and caregiver.

Some hospitals have developed in-chart interventions to address this issue. The Barrow Institute in Arizona and Hutt Hospital in New Zealand implemented EMR notices that alerted prescribing providers if a contraindicated medication was ordered for a patient with PD (Aslam et al., 2020; Lance et al., 2021). The Barrow Institute decreased contraindicated medication use from 42.5 to 17.5% while Hutt Hospital reduced contraindicated medication use from 33 to 5% and reduced length of stay (LOS) by 50%.

Reducing in-hospital complications and LOS was even more important in the COVID-19 era where resources were often limited. In a preliminary data analysis at our institution, 24% of hospitalized PD patients received a contraindicated medication. While this was at the lower end of the above referenced range of contraindicated medication administration at other hospitals, it left plenty of room for improvement. The aims of this study were to determine the effects of contraindicated medication administration in PD patients on ED and hospital outcomes and to develop a tool to reduce contraindicated medication use for PD patients by alerting providers of a possible drug-disease interaction.

## Materials and methods

### Approvals and research protections

This study was determined to be exempt by the Colorado Multiple Institutional Review Board (COMIRB) at the University of Colorado Anschutz Medical Campus.

### Study design

**Figure 1:**
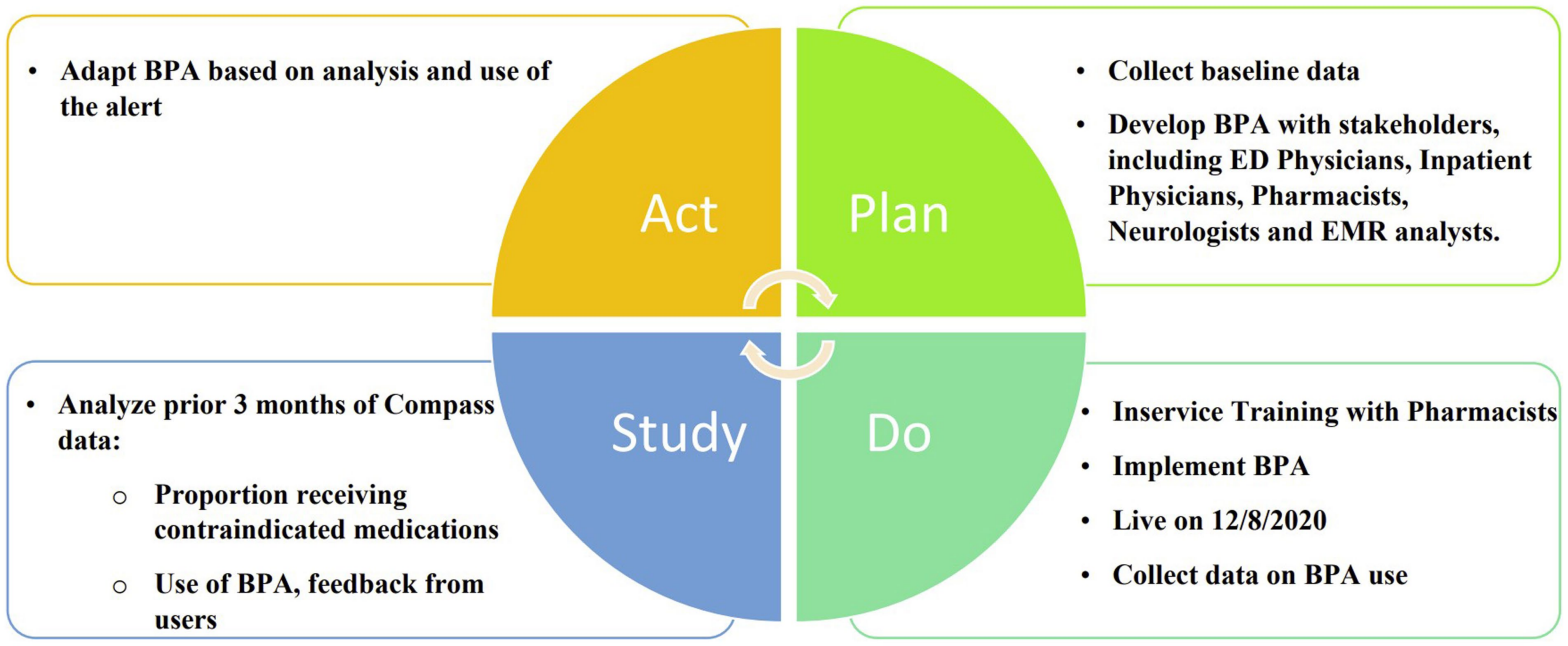
Development and implementation of best practice alerts using PDSA Cycles.

**Figure 2:**
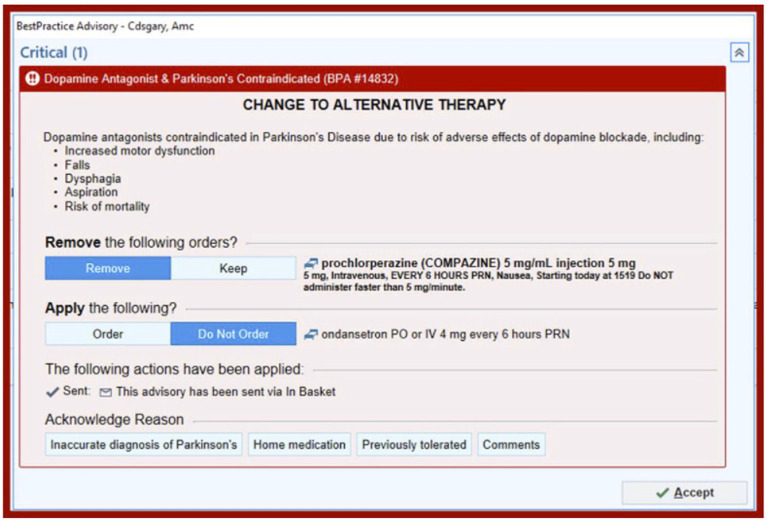
Best practice alert for nausea as the indication for the medication order.

In this prospective cohort study, a best practice alert (BPA) was developed and implemented using a quality improvement “Plan, Do, Study, Act” (PDSA) cycle (Figure 1). It was incorporated into Epic, the electronic medical record (EMR) system at the University of Colorado Hospital. The alert was designed to be triggered when a patient with a diagnosis of Parkinson’s disease, identified by ICD-10 code “G20” in their problem list or past medical history, was prescribed an antidopaminergic medication in the Emergency Department or in the hospital. The BPA warned that dopamine antagonists were contraindicated in Parkinson’s disease and listed several possible adverse effects as shown in Figure 2. Prescribers were given the option to remove the order, keep the order, or apply a safe alternative that was provided in the alert. In order to provide appropriate alternatives, four versions of the BPA were created for different indications. These included “nausea,” “agitation,” “promotility agent,” and “other.” Depending on the indication for the order, appropriate alternatives would be suggested (eg, quetiapine instead of olanzapine for agitation; ondansetron instead of prochlorperazine for nausea). If the order was kept, an acknowledged reason was required, including “inaccurate diagnosis of PD,” “home medication,” and “previously tolerated.” The inpatient pharmacist was notified if the contraindicated medication was ordered, and they were instructed to reach out to the prescriber to discuss the order. Patients were excluded if they had a diagnosis of a secondary or drug-induced parkinsonism, identified by the ICD-10 code “G21”.

Prior to implementation of the BPA, in-service trainings were held with the inpatient pharmacists to discuss the BPAs and their role in discussing the orders and alternatives with providers who prescribed a contraindicated medication.

Using the same inclusion and exclusion criteria, a retrospective chart review was performed to determine baseline rates of contraindicated medication use for patients with Parkinson’s disease seen in the Emergency Department or hospital and were compared to the 12 months post-BPA.

### Study outcomes

The primary outcomes were receipt of a contraindicated medication, length of stay (LOS) and readmission within 30 days. We also collected data on admitting diagnosis, admitting service, neurology involvement, age, gender, race, and ethnicity. For the first 6 months, we also collected data on how often the BPA was triggered, the number of times it was overridden, how often an alternative from the BPA was given and the number of times the contraindicated medication as given.

### Statistical analysis

#### Statistical methods

Frequency and percentages were calculated for categorical variables, and mean and standard deviation were calculated for continuous variables. Summary statistics were broken down by time period (pre- and post-BPA), and by encounter type (ED Visit or Admission). Patient demographics were assessed on unique patients, while treatment characteristics were assessed at the encounter level. Binary outcomes were analyzed with relative risk models, with generalized estimating equations (GEE) accounting for repeated measures. Length of stay was analyzed with Cox proportional hazards models. All tests were two-sided and statistical significance was set at *p* < 0.05 unless otherwise noted. Statistical analyses were performed using the SAS statistical software package version 9.4 (SAS Institute Inc., Cary, NC).

## Results

In the 12 months prior to BPA implementation, a total of 318 ED visits and 229 inpatient admissions occurred for patients with a diagnosis of Parkinson’s disease (Table 1). Post-intervention, there were a total of 317 ED visits and 266 inpatient admissions. There were no differences in age, gender, race, and ethnicity between the pre- and post-intervention groups. The majority of the patients were white and non-Hispanic. The mean age was 72–73 years old and about 60% were male.

**Table 1:** Characteristics of PD patients who received care in the Emergency Department or Hospital Pre and Post Intervention.

	Pre-BPA		Post-BPA	
	ED Visit *N* = 176 patients, 318 encounters	Admissions *N* = 147 patients, 229 encounters	ED Visit *N* = 164 patients, 317 encounters	Admissions *N* = 160 patients, 266 encounters
Mean age, SD (years)	72.40 (11.19) (first encounter)	72.22 (11.45) (first encounter)	72.79 (10.62) (first encounter)	73.03 (10.29) (first encounter)
Sex, *N* (percent male)	96 (54.55%)	93 (63.27%)	88 (53.66%)	105 (65.63%)
Race				
White	137 (77.84%)	120 (81.63%)	132 (80.49%)	130 (81.25%)
Black	21 (11.93%)	9 (6.12%)	8 (4.88%)	10 (6.25%)
Asian	5 (2.84%)	6 (4.08%)	2 (2.50%)	4 (2.50%)
Other	10 (5.68%)	9 (6.12%)	15 (9.15%)	11 (6.88%)
Multiple race	3 (1.70%)	3 (2.04%)	7 (4.27%)	5 (3.13%)
Ethnicity				
Non-Hispanic	162 (92.05%)	136 (92.52%)	139 (84.76%)	145 (90.63%)
Hispanic	14 (7.95%)	10 (6.80%)	25 (15.24%)	15 (9.38%)
Patient refused	0 (0.00%)	1 (0.68%)	0 (0.00%)	0 (0.00%)
Neurologist involved (*N*, percent yes)	Patient: 16 (9.09%) (at least once) Encounter: 17 (5.35%)	Patient: 57 (38.78%) (at least once) Encounter: 73 (31.88%)	Patient: 11 (6.71%) (at least once) Encounter: 17 (5.35%)	Patient: 53 (33.13%) (at least once) Encounter: 73 (31.88%)
Length of stay, Mean, SD (days)	Patient total: 0.40 (0.63) Encounter: 0.22 (0.42)	Patient total: 8.78 (9.78) Encounter: 5.64 (5.71)	Patient total: 0.47 (0.80) (N = 163) Encounter: 0.25 (0.48) (N = 311)	Patient total: 9.04 (10.63) Encounter: 5.44 (6.09)
Mean # of encounters per PD patient, SD	1.81 (1.44)	1.56 (0.74)	1.93 (1.76)	1.66 (1.01)

**Figure 3:**
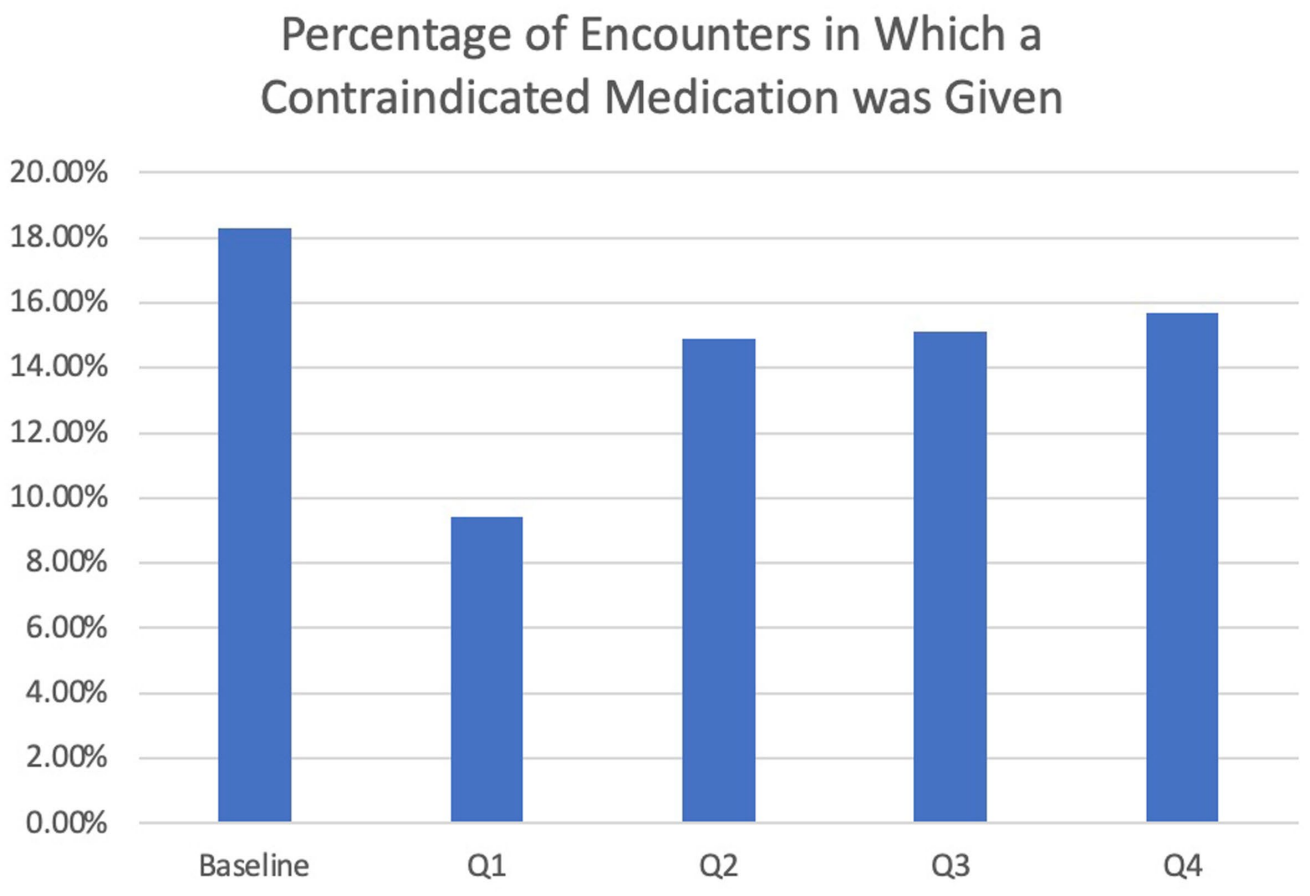
Percentage of encounters in which a contraindicated medication was given by time period.

The overall rate of contraindicated medication use was low for ED visits. In the encounters for 12 months pre-intervention, 4.7% of ED encounters involved the use of a contraindicated medication compared to 5.8% of 12 months post-intervention encounters, which was not statistically significantly different (*p* = 0.55). Regarding inpatient encounters, 18.3% involved the use of a contraindicated medication pre-intervention compared to 13.3% post-intervention for a 27.3% improvement (Table 2). However, there was a larger reduction (48.6%) in the use of contraindicated medications for inpatient admissions in the first 3 months post-intervention versus the first 12 months post-intervention (9.4% vs. 18.3%), after which the use of antidopaminergic medications increased again (Figure 3). During this 3-month period, the BPA was triggered 57 times and overridden 21 times. The inpatient pharmacists approved the use of medications 11 times, most commonly because it was a home medication, the diagnosis of Parkinson’s disease was in question, or the medication was previously tolerated. After the first 3 months, the BPA was overridden more often at 31 times. In addition, there were several instances in which the pharmacist on shift did not see the alert, leading to more antidopaminergic orders getting approved.

**Table 2:** Frequency of contraindicated medication use by encounter type, pre and post intervention.

	Pre-BPA		Post-BPA	
	ED visit *N* = 176 patients, 318 encounters	Admissions *N* = 147 patients, 229 encounters	ED Visit *N* = 164 patients, 317 encounters	Admissions *N* = 160 patients, 266 encounters
Number (%)	Patients: 15 (8.52%) (at least once) Encounters: 15 (4.72%)	Patients: 37 (25.17%) (at least once) Encounters: 42 (18.34%)	Patients: 16 (9.76%) (at least once) Encounters: 18 (5.68%)	Patients: 24 (15.00%) (at least once) Encounters: 36 (13.53%)

Receipt of a contraindicated medication was associated with a longer length of stay both pre- and post-intervention (Table 1). This outcome was analyzed using hazard ratios. Pre-intervention, the contraindicated medication administration reduced the hazard of leaving the hospital (increased length of stay) with a hazard ratio of 0.62 (*p* = 0.0005). Post-intervention, the findings were similar, with a hazard ratio of 0.64 (*p* = 0.0065). There was no significant difference in mean or median length of stay pre- versus post-intervention. Similarly, there was an increase in 30-day readmission rates with receipt of a contraindicated medication versus not, although the difference was marginally statistically non-significant with a risk ratio of 0.69 (*p* = 0.073). There was an increased 30-day readmission rate in patients who received a contraindicated medication post-intervention with a risk ratio of 1.84 (*p* = 0.0001). There was no difference in readmission rates pre- and post-intervention in patients who did not receive contraindicated medications.

**Figure 4:**
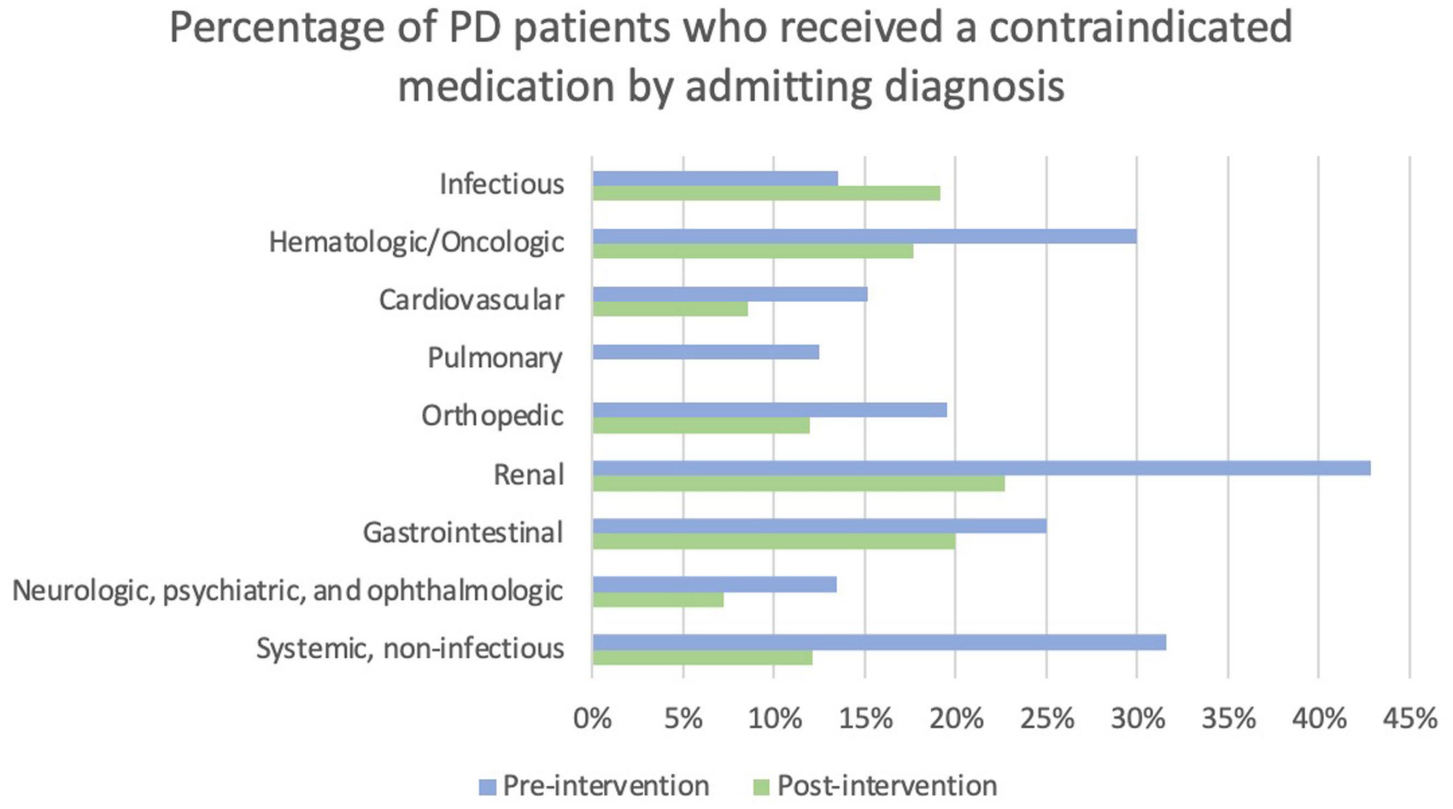
Percentage of PD patients who received a contraindicated medication by admitting diagnosis. Note that no patients admitted with a pulmonary diagnosis received a contraindicated medication post-intervention.

Admitting service and admitting diagnosis were evaluated in the context of whether patients received a contraindicated medication. Patients admitted to a surgery service were more likely to receive a contraindicated medication than patients admitted to a neurology service (22.9% versus 6.9%) post-intervention; however, the omnibus tests were not statistically significant. The percentage of patients who received a contraindicated medication pre- and post-intervention by admitting diagnosis category is displayed in Figure 4. Statistical analysis of this data was limited by low sample sizes in some categories and missing admitting diagnosis in the EMR in many patients.

Neurologist involvement in patient care was assessed by presence or absence of a neurologist’s note during the encounter. Pre-intervention, there was a risk ratio of 0.58 (*p* = 0.078) for administration of a contraindicated medication with neurologist involvement versus without. Post-intervention, the risk ratio was 1.14 (*p* = 0.58). While there was a trend toward lower risk of the use of a contraindicated medication when a neurologist was involved in the care of the patient pre-intervention, it was not statistically significant. This analysis is also limited by not accounting for if the neurologist was involved before or after the receipt of a contraindicated medication.

The most commonly prescribed contraindicated medications both pre- and post-intervention are displayed in Table 3.

**Table 3:** Contraindicated medications by frequency of receipt.

	Pre-intervention (12 months)		Post-intervention (12 months)	
Medication	Frequency*	Percent	Frequency*	Percent
Prochlorperazine	16	2.93	11	1.89
Olanzapine	14	2.56	22	3.77
Hydroxyzine	9	1.65	10	1.72
Haloperidol	8	1.46	6	1.03
Metoclopramide	6	1.10	6	1.03
Promethazine	6	1.10	1	0.17
Aripiprazole	1	0.73	2	0.34
Risperidone	3	0.55	4	0.69
Chlorpromazine	1	0.18	0	0.00
Ziprasidone	0	0.00	1	0.17
Total	64	NA	63	NA

*Number of ER and admissions in which the medication was given at least once.

## Conclusion and discussion

In this study, we developed and implemented a tool to alert prescribing providers about potential drug-disease interactions and successfully reduced the administration of contraindicated medications, with the most significant impact observed in the first 3 months after the tool’s release. In addition, we found that increased LOS and 30-day re-admission rates were associated with contraindicated medication use.

Our BPA decreased the rate of contraindicated medication administration in admitted patients with Parkinson’s disease. In the first 3 months post-intervention, the rate of administration of these agents nearly halved. While the rate of administration continued to trend lower than baseline in the subsequent 9 months, the reduction was much more modest. The first 3 months were also associated with a higher pharmacist involvement and prescriber responsiveness to the alerts, likely accounting for the larger reduction in contraindicated medication use during this time period. As with any repetitive alerting system, there is a potential for practitioners to become fatigued from frequent reminders. This phenomenon could decrease the tool’s efficacy over time, resulting in reduced use and possibly undermining its benefits. To address the issue of alert fatigue, several strategies could be employed to increase the engagement of both prescribers and pharmacists. Firstly, periodic education and retraining sessions can serve as timely reminders about the importance of the tool and its impact on patient outcomes. Implementing personalized feedback mechanisms to monitor individual prescriber and pharmacist performance may also encourage continued usage of the tool, as successfully executed by Raja et al. (2015).

By providing a tangible solution to the prescription of contraindicated medications in hospitalized Parkinson’s patients through the development of a tool, we contribute to the growing body of research on improving medication safety in neurodegenerative disorders. The successful implementation of the tool adds to the literature regarding the potential for technology-based interventions to improve patient care and reduce complications in vulnerable populations. This study replicates findings in the literature of a BPA’s ability to reduce administration of contraindicated medications and the association of receipt of a contraindicated medication with length of stay. The study adds to the literature data from a facility with lower-than-average baseline administration of contraindicated medications, suggesting that a BPA is an effective tool in this setting.

Our findings reveal a significant association between the administration of contraindicated medications and increased length of stay and 30-day readmissions in PD patients. By reducing the use of contraindicated medications through the implementation of a BPA, both length of stay and 30-day readmissions can be reduced. This is important for healthcare providers and institutions, as reducing both length of stay and readmissions is important for both optimizing healthcare resources and for improving patient outcomes.

The statistically significant difference between pre- and post-intervention 30-day readmission rates for patients who received contraindicated medications may be due to alternative medications suggested by the BPA being reasonable for less acutely ill patients, while the more acutely ill patients required the originally prescribed antidopaminergic therapy.

In conclusion, this study not only emphasizes the importance of avoiding contraindicated medications in PD patients but also illustrates the efficacy of a well-designed tool in reducing the occurrence of such medication errors, even in facilities where baseline administration of contraindicated medications is lower than average. While alert fatigue remains a potential challenge, proactive strategies to address this issue can sustain the tool’s impact and ensure its continued usage among healthcare providers. Overall, our findings hold valuable implications for enhancing patient safety, optimizing hospital care, and promoting medication management practices in Parkinson’s disease and other neurodegenerative disorders.

## Data availability statement

The raw data supporting the conclusions of this article will be made available by the authors, without undue reservation.

## Ethics statement

The studies involving humans were approved by the Colorado Multiple Institution Review Board. The studies were conducted in accordance with the local legislation and institutional requirements. Written informed consent for participation was not required from the participants or the participants’ legal guardians/next of kin in accordance with the national legislation and institutional requirements.

## Author contributions

CG: Methodology, Writing – original draft, Writing – review & editing. SS: Methodology, Writing – review & editing. EW: Writing – review & editing. JB: Writing – review & editing. RC: Writing – review & editing. KT: Writing – review & editing. MF: Methodology, Writing – original draft, Writing – review & editing.
